# Impact of COVID‐19 Vaccination on Menstrual Irregularities, Bleeding Patterns, and Cycle Duration: A Systematic Review and Meta‐Analysis

**DOI:** 10.1002/hsr2.70882

**Published:** 2025-06-16

**Authors:** Ganesh Bushi, Abhay M. Gaidhane, Nasir Vadia, Soumya V. Menon, Kattela Chennakesavulu, Rajashree Panigrahi, Muhammed Shabil, Diptismita Jena, Harish Kumar, Anju Rani, Sanjit Sah, Shivam Rohilla, Mahendra Pratap Singh, Khang Wen Goh

**Affiliations:** ^1^ Chitkara Centre for Research and Development Chitkara University Himachal Pradesh India; ^2^ School of Pharmaceutical Sciences Lovely Professional University Phagwara India; ^3^ Jawaharlal Nehru Medical College, and Global Health Academy, School of Epidemiology and Public Health Datta Meghe Institute of Higher Education Wardha India; ^4^ Marwadi University Research Center, Department of Pharmaceutical Sciences, Faculty of Health Sciences Marwadi University Rajkot Gujarat India; ^5^ Department of Chemistry and Biochemistry, School of Sciences JAIN (Deemed to be University) Bangalore Karnataka India; ^6^ Department of Chemistry Sathyabama Institute of Science and Technology Chennai Tamil Nadu India; ^7^ Department of Microbiology, IMS and SUM Hospital Siksha ‘O’ Anusandhan (Deemed to be University) Bhubaneswar Odisha India; ^8^ University Center for Research and Development Chandigarh University Mohali Punjab India; ^9^ Centre of Research Impact and Outcome Chitkara University Rajpura Punjab India; ^10^ Division of Research and Innovation Uttaranchal University Dehradun India; ^11^ New Delhi Institute of Management New Delhi India; ^12^ Department of Microbiology Graphic Era (Deemed to be University) Dehradun India; ^13^ Graphic Era Hill University, Clement Town Dehradun India; ^14^ Department of Pediatrics, Dr. D. Y. Patil Medical College, Hospital and Research Centre Dr. D. Y. Patil Vidyapeeth (Deemed‐to‐be‐University) Pune Maharashtra India; ^15^ Department of Public Health Dentistry, Dr. D. Y. Patil Medical College Hospital and Research Centre Dr. D. Y. Patil Vidyapeeth (Deemed‐to‐be‐University) Pune Maharashtra India; ^16^ SR Sanjeevani Hospital Kalyanpur Siraha Nepal; ^17^ Noida Institute of Engineering and Technology Greater Noida India; ^18^ Center for Global Health Research, Saveetha Medical College and Hospital Saveetha Institute of Medical and Technical Sciences, Saveetha University Chennai India; ^19^ Faculty of Data Science and Information Technology INTI International University Nilai Malaysia; ^20^ Faculty of Mathematics and Natural Sciences Universitas Negeri Padang Padang Indonesia

**Keywords:** adverse effects, COVID‐19 vaccines, menstrual cycle, menstruation disturbances, reproductive health, vaccination, women's health

## Abstract

**Background:**

COVID‐19 vaccination has raised concerns regarding its potential effects on women's reproductive health, particularly menstrual irregularities. This systematic review and meta‐analysis aimed to assess the impact of COVID‐19 vaccination on menstrual disturbances, bleeding patterns, and cycle duration among women of reproductive age.

**Methods:**

A systematic search of PubMed, Embase, and Web of Science was conducted up to April 11, 2025. The study protocol was registered with the PROSPERO (CRD42024500832). Studies reporting menstrual changes postvaccination in women aged 13–50 were included. Data extraction and quality assessment were performed independently by two reviewers. Meta‐analyses using random‐effects models were conducted in R (version 4.3), with heterogeneity assessed using the *I*² statistic.

**Results:**

Out of 586 records, 43 studies comprising 747,763 women met the inclusion criteria. The pooled RR for menstrual disturbances in vaccinated versus unvaccinated women was 1.03 (95% CI: 0.67–1.57; *p* = 0.88), indicating no significant association. Excluding one outlier increased the RR to 1.14 (95% CI: 0.97–1.34; *p* = 0.08). The overall pooled prevalence of menstrual disturbances postvaccination was 34% (95% CI: 26%–43%). Among vaccinated women, lighter bleeding was reported in 12.6%, heavier bleeding in 15.1%, irregular menstruation in 19.0%, and regular cycles in 56.6%. Shortened cycles occurred in 8.5%, longer cycles in 9.3%, amenorrhea (≥ 24 days) in 9.2%, and infrequent cycles (> 38 days) in 11.0%. All analyses showed high heterogeneity (I² = 98%–100%). Sensitivity analyses confirmed the robustness of findings, though Egger's test indicated potential publication bias (*p* = 0.0384).

**Conclusion:**

COVID‐19 vaccination was not significantly associated with an increased risk of menstrual disturbances. Although minor changes such as altered bleeding patterns and cycle length were observed in some women, the overall impact on menstrual health was minimal.

## 1 Introduction

The rapid global dissemination and development of COVID‐19 vaccines in response to the pandemic have represented a watershed moment in the annals of public health [1]. As the world grappled with the relentless spread of the SARS‐CoV‐2 virus, which precipitated a global health crisis of unprecedented scale, the introduction of vaccines emerged as a beacon of hope [2]. By 2024, the international community will have achieved a remarkable feat of distributing over 13.53 billion doses of COVID‐19 vaccines, achieving a coverage rate of 70.6% of the global population with at least one dose [3, 4]. This extensive vaccination effort has been instrumental in mitigating the transmission of the virus and reducing the severity of disease outcomes, thereby curtailing the pandemic's impact on human life [5].

Despite the success of global vaccination campaigns, rare but serious adverse events have emerged, warranting continued safety monitoring. Anaphylaxis, estimated to occur at approximately 5 cases per million doses administered, has been documented [6]. Other rare conditions, such as Guillain‐Barré syndrome (GBS) and thrombotic thrombocytopenic purpura (TTP)—neurological and hematological disorders, respectively—have also been reported [7]. Although these adverse events are rare, with estimated incidences of 1 to 2 cases per million doses for GBS and 1 to 10 cases per million for TTP, they are hypothesized to share immunological mechanisms that may also contribute to menstrual irregularities. Specifically, vaccine‐induced immune or inflammatory responses may transiently affect the hypothalamic‐pituitary‐ovarian axis or trigger endometrial changes [8]. European surveillance, via EudraVigilance, reported 16,422 cases for Comirnaty and 2840 for Spikevax by early 2022, prompting the EMA to add heavy menstrual bleeding as a side effect of unknown frequency on October 28, 2022, based on a possible causal link, though most cases were nonserious and temporary [9, 10]. Several observational studies have reported temporary menstrual changes postvaccination, including heavier bleeding, delayed menstruation, or changes in cycle length, in approximately 10%–20% of vaccinated women [11, 12]. These menstrual disturbances are generally short‐lived and resolve within 1 to 2 cycles [13, 14]. Importantly, no causal link has been established between COVID‐19 vaccination and either menstrual irregularities or rare conditions such as GBS and TTP [15].

The potential impact of COVID‐19 vaccination on women's reproductive health has become a growing concern and topic of scientific and public discourse. Menstrual regularity, a vital sign of reproductive and overall health, has become central to discussions surrounding vaccine safety [16, 17]. Concerns about possible associations between vaccination and adverse reproductive outcomes—such as infertility, pregnancy loss, and menstrual cycle disturbances—have led to increased scientific inquiry [18]. While large‐scale studies have generally found no significant association between COVID‐19 vaccines and adverse reproductive outcomes, self‐reported data and anecdotal evidence of menstrual changes have contributed to uncertainty and public concern [19, 20].

To address this important issue and inform public health policy, a comprehensive and systematic evaluation of the available evidence is needed. This systematic review and meta‐analysis aimed to assess the impact of COVID‐19 vaccination on menstrual cycle characteristics and outcomes among women of reproductive age. By synthesizing findings from both qualitative and quantitative studies, this review sought to clarify potential associations and address gaps in current knowledge regarding the vaccine's reproductive safety profile.

## 2 Methods

This systematic review and meta‐analysis rigorously followed the guidelines set by PRISMA (Supporting Information S1: Table S1). The study protocol was officially registered with the PROSPERO (CRD42024500832). We utilized the Nested Knowledge web application for efficient screening and data extraction (Nested‐Knowledge, MN, USA).

### 2.1 Eligibility Criteria

We focused on studies evaluating the impact of COVID‐19 vaccination on women's reproductive health, particularly changes in menstrual cycles. The inclusion criteria were observational studies (cohort, cross‐sectional, case‐control) with clear definitions and measurements of vaccination status and outcomes related to reproductive health published in English. Exclusion criteria were studies without precise definitions of vaccination, participants with pre‐existing menstrual disorders, those involving populations under 18 or over 50 years of age, and nonempirical articles, such as reviews or editorials. A detailed list of inclusion criteria is provided in Supporting Information S1: Table S2.

### 2.2 Search Strategy

A comprehensive search was conducted across PubMed, Embase, and Web of Science up to April 11, 2025. using terms related to “COVID‐19 vaccination” and “Menstruating women” or “menstruation”. This search was unfiltered by date to encompass the entire range of available literature and supplemented by manual searches of references in the selected studies and key reviews for completeness (Supporting Information S1: Table S3).

### 2.3 Screening

Articles were screened by two independent reviewers using Nested Knowledge software. They began by screening the titles and abstracts to eliminate irrelevant studies, followed by full‐text reviews of potentially relevant articles. In cases of disagreement, a third independent reviewer was consulted to resolve discrepancies and guarantee a comprehensive and impartial selection process for the systematic review. The initial screening of titles and abstracts independently assessed study eligibility, and any differences were resolved through discussion or a third reviewer. Full‐text reviews determined inclusion based on established criteria and employed a Nested Knowledge platform for systematic evaluation.

### 2.4 Data Extraction

Data extraction was independently performed by two reviewers, and accuracy was verified by a third reviewer. The information extracted included the study design, individual demographic details, sample size, country, and number of individuals experiencing irregular or regular menstruation post‐COVID‐19 vaccination, focusing on the effects of the vaccine on reproductive health. This included details on the total number of patients and those who developed menstrual disturbances post‐COVID‐19 vaccination. To assess associations, we recorded the totals in both the vaccinated and non‐vaccinated groups, along with the number of events, specifically in patients who developed menstrual disturbances. The “tagging” feature in Nested Knowledge was employed for efficient data extraction, and the data were organized in Microsoft Excel. Any discrepancies in data extraction were resolved through discussion or consultation with a third reviewer.

### 2.5 Quality Assessment

The Modified Newcastle–Ottawa Scale (NOS) was employed to evaluate the quality of the studies included in this review [21]. The methodological quality and risk of bias of the observational studies were assessed using a modified version of the NOS. Tailored for non‐randomized studies, this scale evaluates studies based on criteria related to group selection, sample size, and outcome determination. The key factors considered included the representativeness of cross‐sectional studies, the accuracy of exposure measurement, and the quality of outcome reporting. With a maximum achievable score of six points, the NOS score serves as an indicator of the overall quality of each study. Studies were classified as high quality if they scored 5–6 points, moderate quality if they scored 3–4 points, and low quality if they scored less than 3 points.

### 2.6 Data Synthesis

All statistical procedures aimed to provide a comprehensive and reliable overview of the effects of post‐COVID‐19 vaccination on menstrual health outcomes using R software version 4.3 [22]. Heterogeneity among studies was assessed using the *I*² statistic, applying a random‐effects model for *I*² > 50% to account for potential variations [23, 24]. We included 95% prediction intervals to forecast the effect ranges in future studies and conducted sensitivity analyses to ensure the reliability of the results, with statistical significance set at *p* < 0.05. In the meta‐analysis, we calculated Risk ratio (RR) from both vaccinated and non‐vaccinated groups to assess the RR of menstrual disturbances, employing a random‐effects model to better accommodate study variations. Heterogeneity was assessed quantitatively using the *I*² statistic, where values of > 50% suggested significant heterogeneity. Prediction intervals can be used to forecast the range of effects in future studies [25]. Funnel plot analysis and Egger's test were used to evaluate publication bias. A leave‐one‐out method in the sensitivity analysis helped gauge the impact of single studies on the collective results.

## 3 Results

### 3.1 Literature Search

**Figure 1:**
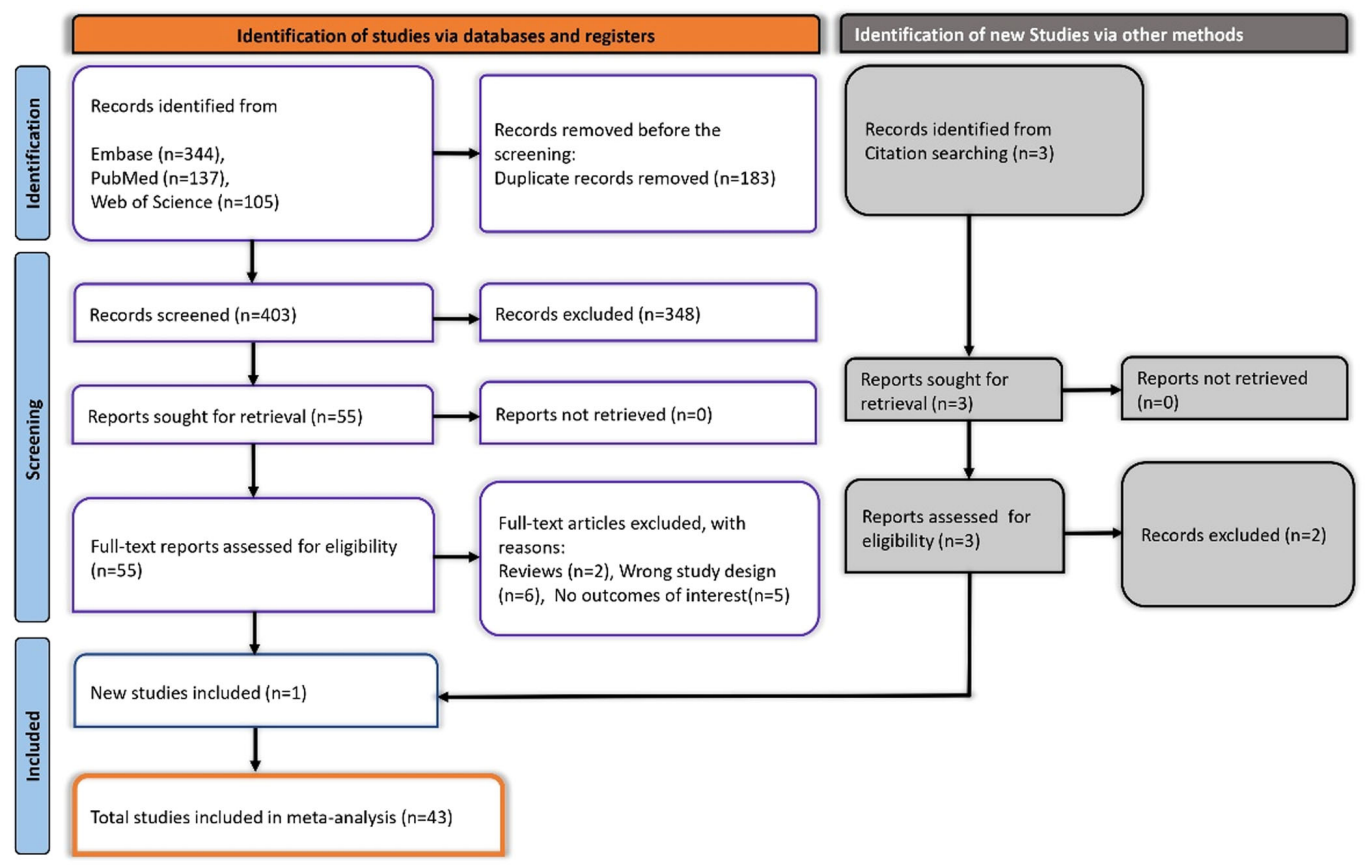
PRISMA flow diagram represents the screening and selection process.

Our literature search across the PubMed, Embase, and Web of Science databases yielded 586 records. After the removal of 183 duplicates, 403 records remained for screening. Titles and abstracts were screened, and 348 studies were excluded as they did not meet the inclusion criteria. We then assessed the full texts of the remaining 55 studies for eligibility. Of these, 13 studies were excluded for reasons including being reviews (*n* = 2), having the wrong study design (*n* = 6), or lacking outcomes of interest (*n* = 5). Additionally, three studies were identified through citation searching, of which one met the eligibility criteria. In total, 43 studies were included in the meta‐analysis. The PRISMA flowchart outlining the literature search and selection process is shown in Figure 1.

### 3.2 Study Characteristics

Table 1 includes 45 studies with a total of 747,763 women, mostly aged between 18 and 53 years, analyzed to investigate the impact of COVID‐19 vaccination on women's reproductive health. The studies covered a wide geographical range, including countries from the Middle East, Europe, North America, South America, Asia, and multi‐country collaborations. They employed various study designs, including retrospective studies [26, 27, 28, 29, 30, 31, 32], cross‐sectional studies [16, 33, 34, 35, 36, 37, 38, 39, 40, 41, 42, 43, 44, 45, 46, 47, 48, 49, 50, 51, 52, 53, 54, 55, 56], case‐control studies [57, 58], and prospective cohort studies [6, 59, 60, 61, 62, 63, 64, 65]. Sample sizes varied considerably, from 79 participants to 666,467 participants. These studies, drawn from a wide array of settings, employed diverse methodologies to explore the association between COVID‐19 vaccination and changes in menstrual patterns, bleeding, and cycle duration. The quality of the included studies, as assessed using the Modified NOS, ranged from 3 to 5, indicating moderate to high methodological quality (Supporting Information S1: Table S4).

**Table 1:** Summary characteristics of included studies.

Study ID	Country	Study design	Age years	Sample size	NOS score
Alahmadi et al. [66]	Saudi Arabia	Retrospective study	18–45 (range)	673	4
Al‐Furaydi et al. [6]	Saudi Arabia	Population‐based cohort	18–45 (range)	338	4
Aljehani et al. [33]	Saudi Arabia	Cross‐sectional study	> 17	535	5
Almomani et al. [34]	Jordan	Cross‐sectional study	18–45 (range)	564	3
Alsaeedi et al. [57]	Saudi Arabia	Case‐control study	NA	79	4
Alvergne et al. [58]	UK	Case‐control study	NA	4989	5
Amer et al. [35]	Egypt, Saudi Arabia, Syria, Libya, and Sudan	Cross‐sectional study	29.6 (mean)	1254	4
Baena‐Garcia et al. [36]	Spain	Cross‐sectional study	31.5 (mean)	14,153	5
Barabas et al. [26]	Hungary	Retrospective study	15–49 (range)	1563	5
Caglayan et al. [59]	Turkey	Prospective cohort study	31–45 (range)	198	4
Dabbousi et al. [37]	Lebanon	Cross‐sectional study	26.9 (mean)	505	4
Darney et al. [60]	UK, USA, and Canada	Observational cohort study	≥ 18	7401	5
Dellino et al. [27]	Italy	Retrospective study	18–45 (range)	100	4
Eskandar et al. [38]	Saudi Arabia	Cross‐sectional study	< 30	1208	3
Farland et al. [61]	USA	Prospective cohort study	≥ 18	545	4
Granese et al. [39]	Italy	Cross‐sectional study	32 (mean)	471	4
Hasdemir et al. [62]	Turkey	Cohort study	35 (mean)	258	4
Issakov et al. [40]	Israel	Cross‐sectional study	≥ 18	7476	3
Jiang et al. [41]	China	Cross‐sectional study	18–53 (range)	869	4
Kareem et al. [42]	Pakistan	Cross‐sectional study	20.67 (mean)	953	3
Khan et al. [28]	Saudi Arabia	Retrospective study	18 (mean)	383	4
Magnus et al. [29]	Norway	Retrospective study	20–40 (range)	666,467	3
Mahfouz et al. [43]	Saudi Arabia	Cross‐sectional study	≥ 18	729	4
Male [30]	UK	Retrospective study	≥ 18	2241	3
Marcelino et al. [44]	Brazil	Cross‐sectional study	≥ 18	1012	5
Matar et al. [45]	Saudi Arabia	Cross‐sectional study	24.02 (mean)	4942	3
Minguez‐Esteban et al. [46]	Spain	Cross‐sectional study	18‐45 (range)	746	4
Muhaidat et al. [47]	Jordan	Cross‐sectional study	34.3 (mean)	2269	4
Parveen [63]	Saudi Arabia	Prospective cohort study	18–45 (range)	154	4
Qashqari et al. [48]	Saudi Arabia	Cross‐sectional study	35.4 (mean)	2338	4
Qazi et al. [49]	India	Cross‐sectional study	15–49 (range)	300	5
Rodriguez Quejada et al. [31]	Continents: North America, South America, Australia, and Europe	Retrospective study	≥ 18	950	4
Safiah et al. [50]	Syrian Arab Republic	Cross‐sectional study	> 18	236	4
Saleh Alzahrani et al. [51]	Saudi Arabia	Cross‐sectional study	≥ 18	1066	5
Sarfraz et al. [52]	Pakistan	Cross‐sectional study	NA	510	4
Sualeh et al. [53]	Pakistan	Cross‐sectional study	≥ 18	384	5
Tandon et al. [54]	India	Cross‐sectional study	20.6 (mean)	773	4
Taşkaldıran et al. [55]	Turkey	Cross‐sectional study	≥ 18	542	4
Toktas et al. [56]	Turkey	Cross‐sectional study	18–50 (range)	586	4
Trogstad et al. [64]	Norway	Cohort study	≥ 18	5765	4
Wali et al. [16]	Saudi Arabia	Cross‐Sectional Study	15–50 (range)	297	5
Wang et al. [65]	USA	Prospective cohort study	33.4 (mean)	3858	4
Zhang et al. [32]	China	Retrospective study	NA	13,118	4

Abbreviations: NA, not available; UK, United Kingdom; USA, United States of America.

### 3.3 Association Between Menstrual Disturbances and COVID‐19 Vaccination

**Figure 2:**
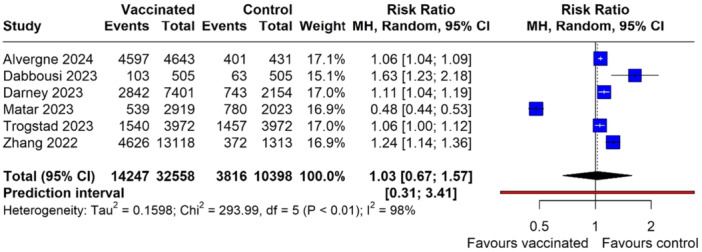
Forest plot depicting the association between menstrual disturbances and COVID‐19 vaccination.

The meta‐analysis included a total of 32,558 vaccinated women, and 10,398 controls showed a slight increase in the risk of menstrual disturbances in vaccinated individuals with a pooled RR of 1.03 (95% CI: 0.67, 1.57) with a *p* = 0.88). The heterogeneity across the included studies was high (*I*² = 98%), and the prediction interval ranged from 0.31 to 3.41, indicating that future studies could report a wide range of effect sizes, from decreased to increased risk of menstrual disturbances, following COVID‐19 vaccination (Figure 2). After identifying “Matar 2023” as an outlier and removing it from the analysis, the recalculated results based on the remaining five studies with 42,956 observations and 18,063 events yielded a pooled RR of 1.14 (95% CI: 0.97; 1.34), with a *p* value of 0.08, indicating that the association was not statistically significant.

### 3.4 Prevalence of Diverse Menstrual Disturbances Following COVID‐19 Vaccination

**Figure 3:**
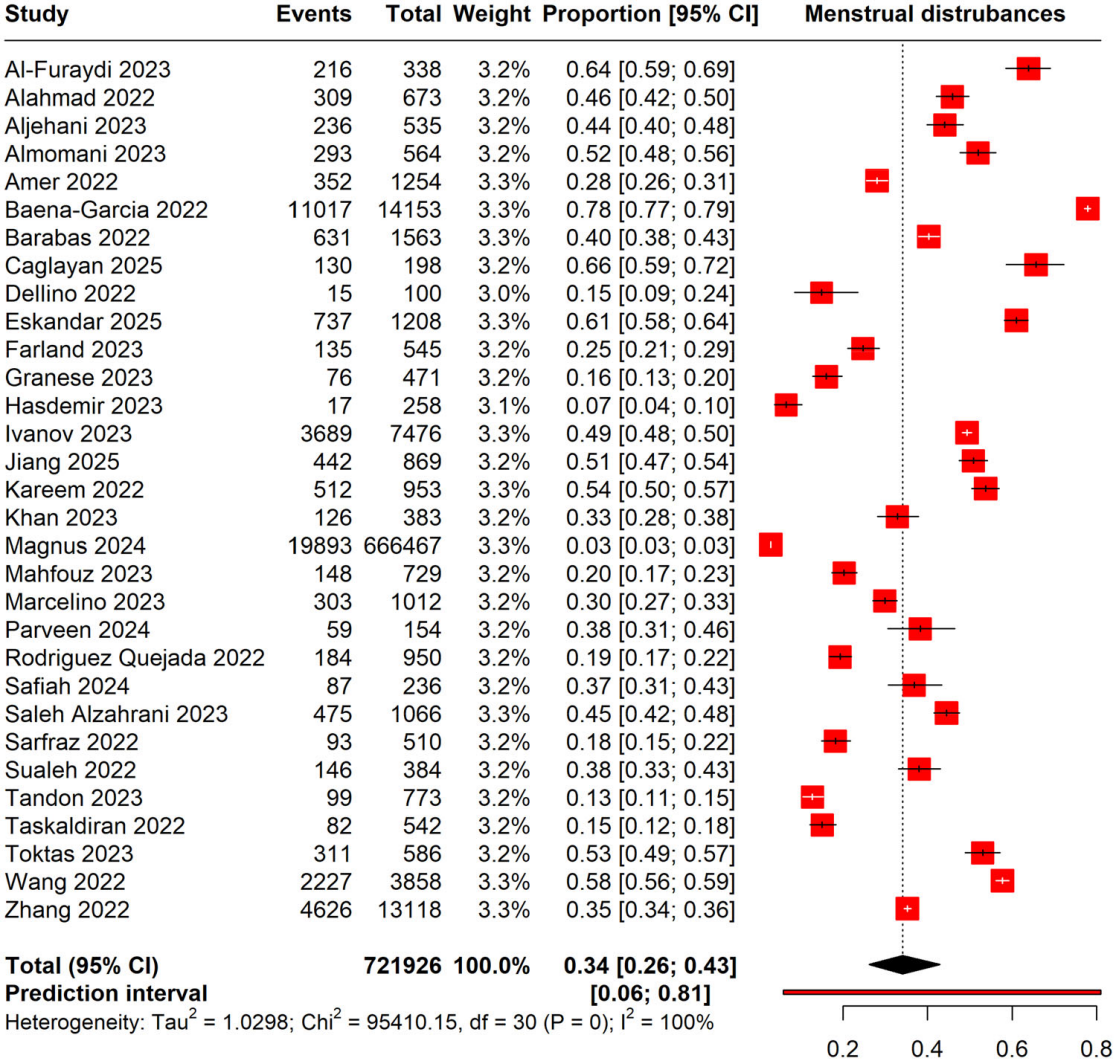
Forest plot illustrating the pooled prevalence of irregular menstruation post‐COVID‐19 vaccination.

A total of 721,926 COVID‐19 vaccinated women were included in the meta‐analysis. The pooled prevalence of menstrual disturbances following vaccination was 34% (95% CI: 26%–43%). The study reporting the highest prevalence was Baena‐Garcia 2022 from Spain, with a rate of 78% (95% CI: 77%–79%). Conversely, the lowest prevalence was reported by Magnus 2024 from Norway, at 3% (95% CI: 3%–3%). Substantial heterogeneity was observed across the studies (*I*² = 100%), indicating significant variability in the reported prevalence rates, likely due to differences in study design, population characteristics, and outcome assessment methods. The prediction interval ranged from 6% to 81%, suggesting that the true prevalence in similar future studies could fall within this range (Figure 3 **)**.

### 3.5 Prevalence of Lighter Bleeding and Heavier Bleeding Post COVID‐19 Vaccination

#### 3.5.1 Lighter Menstrual Bleeding

**Figure 4:**
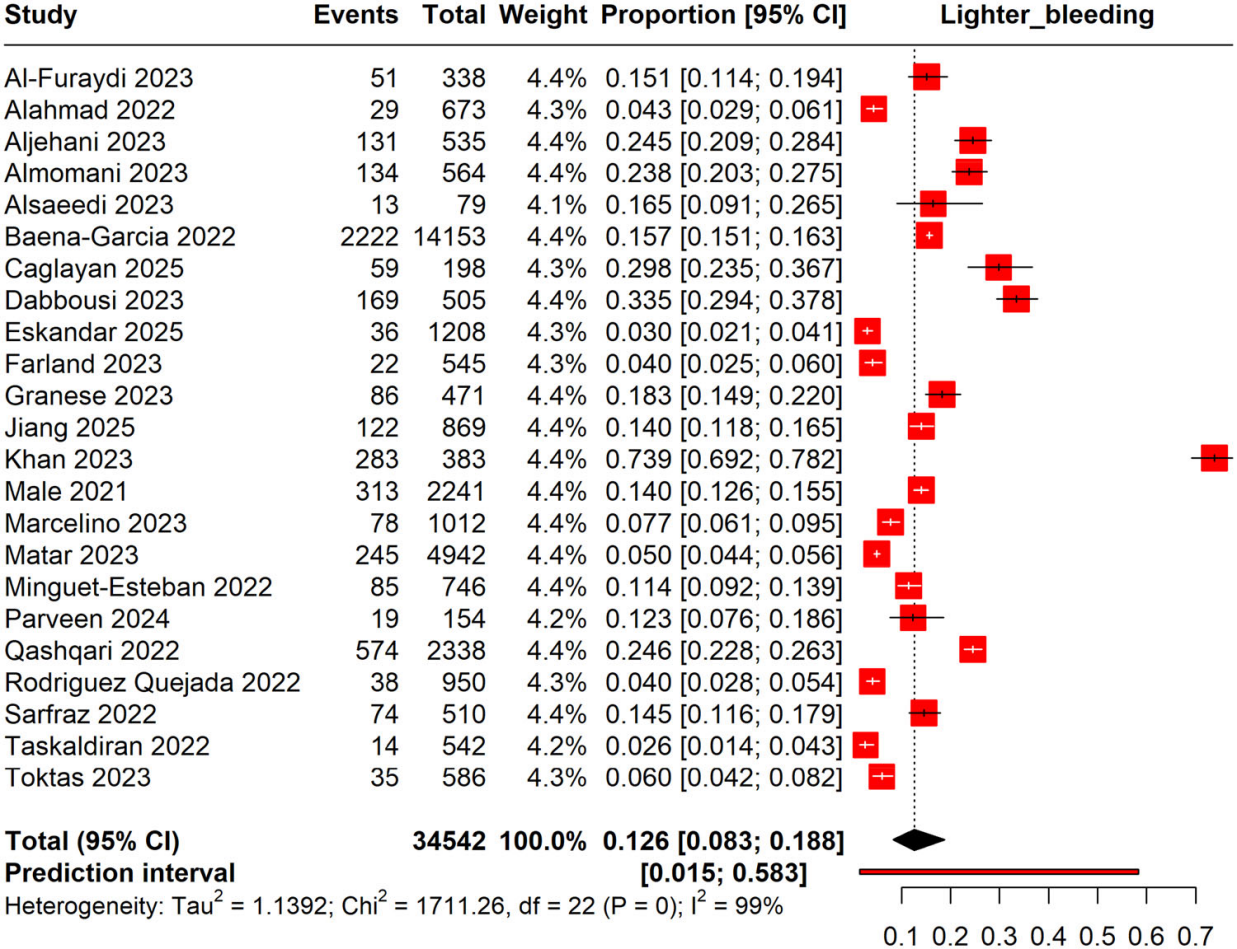
Forest plot depicting the irregular lighter bleeding during menstruation post‐COVID‐19 vaccinations.

The meta‐analysis included 34,542 women and revealed a pooled prevalence of lighter menstrual bleeding following COVID‐19 vaccination to be 12.6% (95% CI: 8.3%–18.8%). The highest reported prevalence was from the study by Khan 2023 at 73.9% (95% CI: 69.2%–78.2%), while the lowest was from Taskaldiran et al. [55] at 2.6% (95% CI: 1.4%–4.3%). The prediction interval ranged from 1.5% to 58.3%, suggesting a wide potential range of true prevalence rates in similar future studies (Figure 4).

#### 3.5.2 Heavier Menstrual Bleeding

**Figure 5:**
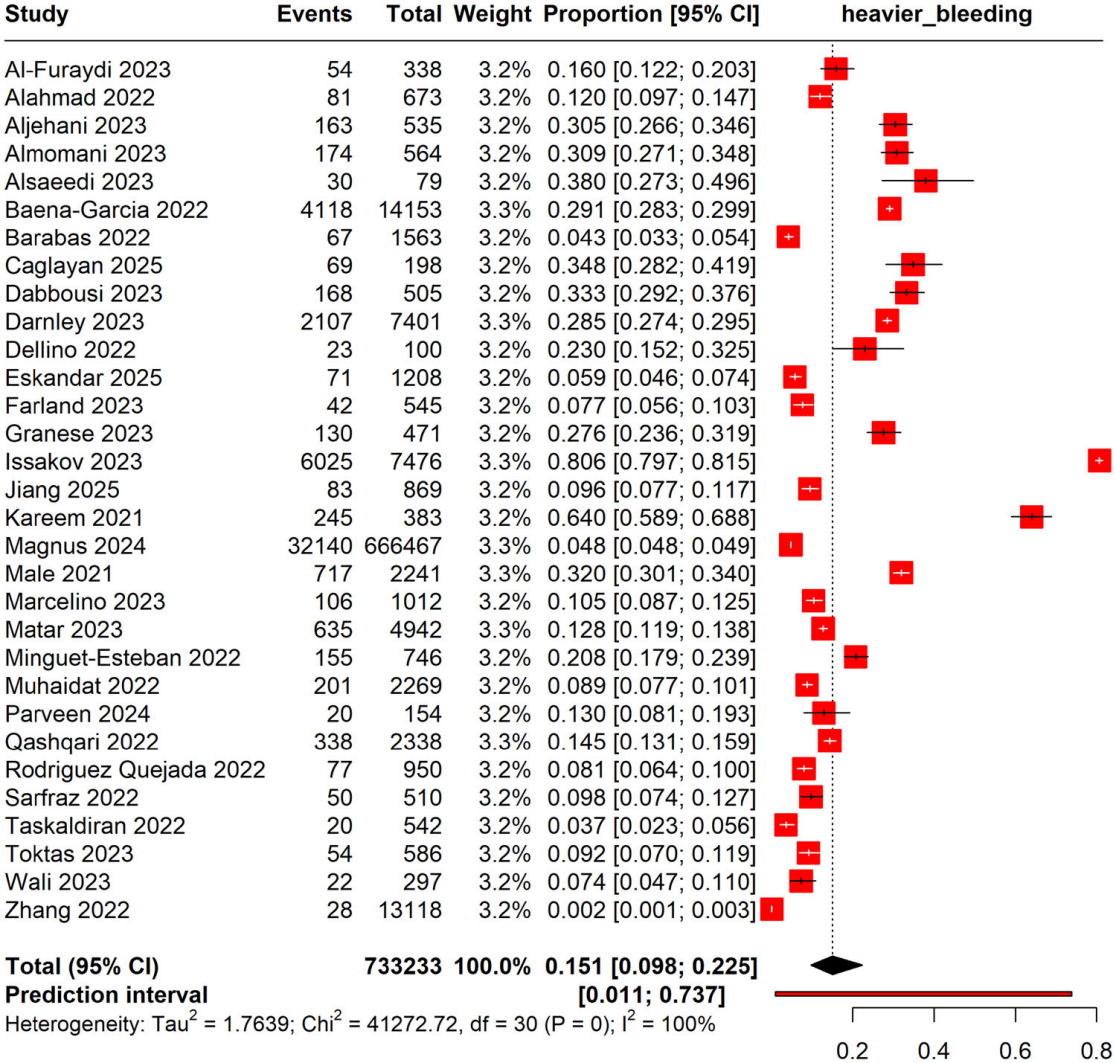
Forest plot showing irregular, heavier bleeding during menstruation post‐ COVID‐19 vaccinations.

The meta‐analysis included 733,233 COVID‐19 vaccinated women and estimated a pooled prevalence of heavier menstrual bleeding at 15.1% (95% CI: 9.8%–22.5%). The highest prevalence was reported by Issakov 2023 at 80.6% (95% CI: 79.7%–81.5%), while the lowest was observed in the study by Zhang et al. [32] at 0.2% (95% CI: 0.01%–0.3%). There was substantial heterogeneity across the included studies (*I*² = 100%), reflecting considerable variability in reported rates, possibly due to differences in geographic populations, assessment methods, and study designs. The prediction interval ranged from 1.1% to 73.7%, indicating a wide potential range for true prevalence in future similar studies (Figure 5).

### 3.6 Regularity of the Menstruation After COVID ‐19 Vaccination

#### 3.6.1 Regular Menstruation

**Figure 6:**
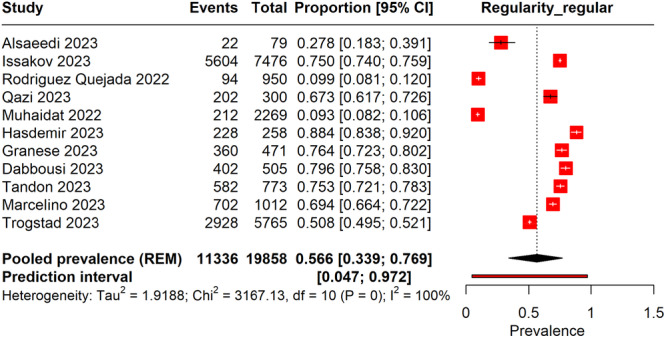
Forest plot representing the prevalence of regular menstrual cycles following COVID‐19 vaccinations.

The pooled prevalence of regular menstruation following COVID‐19 vaccination was 56.6% (95% CI: 33.9%–76.9%), as shown in Figure 6. This indicates that the majority of the participants retained their regular menstrual cycles postvaccination. The heterogeneity across the studies was complete (*I*² = 100%), signifying substantial variability in the reported prevalence rates among different studies. Furthermore, the prediction interval, which ranged from 4.7% to 97.2%, suggested that there could be significant differences in future study outcomes.

#### 3.6.2 Prevalence of Irregular Menstruation

**Figure 7:**
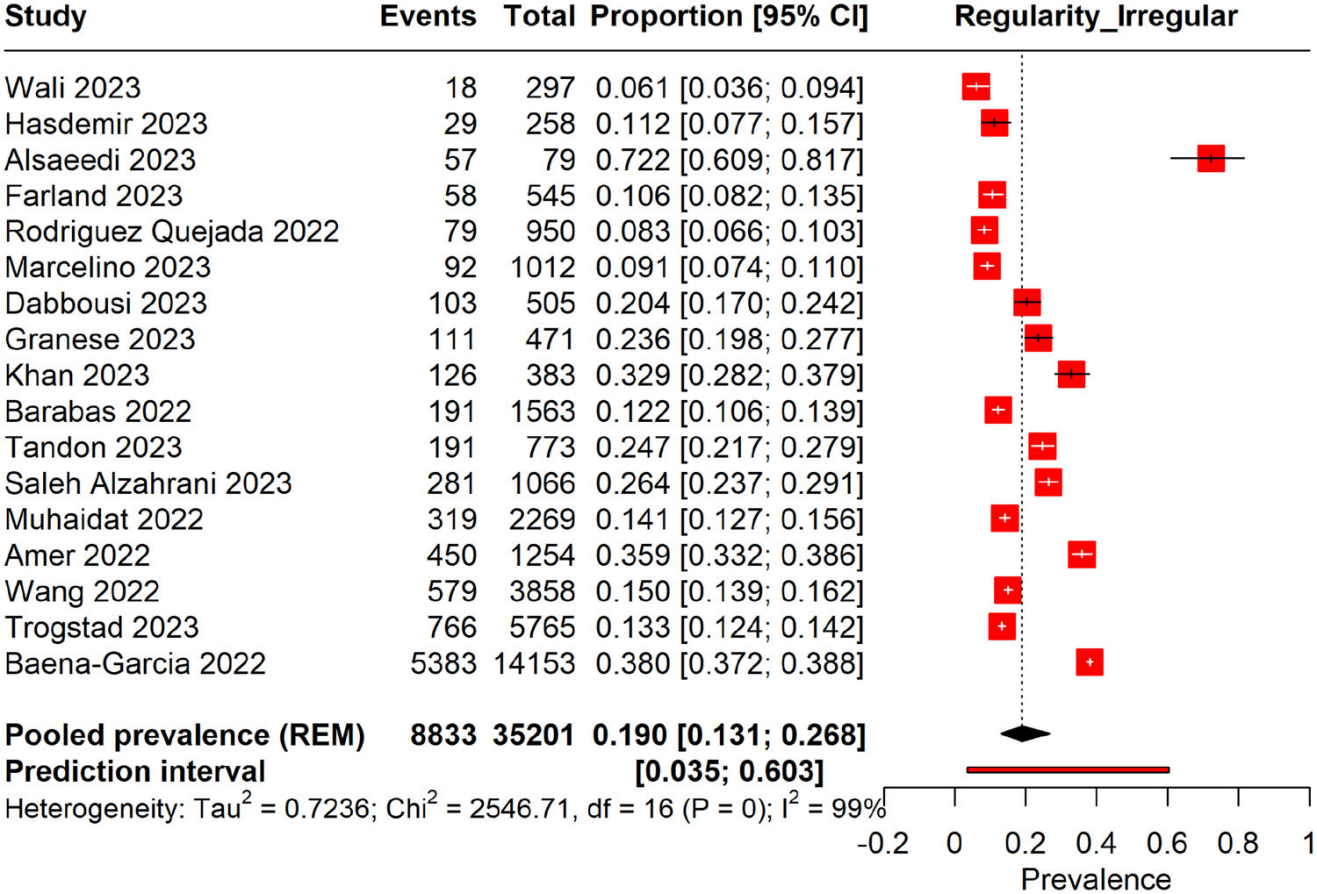
Forest plot representing the prevalence of irregular menstrual cycles following COVID‐19 vaccinations.

Figure 7 Forest plot shows the pooled prevalence of irregular menstruation post‐COVID‐19 vaccination to be 19.0% (95% CI: 13.1%–26.8%), suggesting that approximately one in five women experience irregular menstruation following vaccination. The studies exhibited high heterogeneity (*I*² = 99%), indicating significant variation in prevalence rates across different studies and populations. Additionally, the wide prediction interval, ranging from 3.5% to 60.3%, indicated potential variability in future research findings.

### 3.7 Variations in Menstrual Cycle Duration Subsequent to COVID‐19 Vaccination

#### 3.7.1 Shortened Menstruation Cycles Following COVID‐19 Vaccination

**Figure 8:**
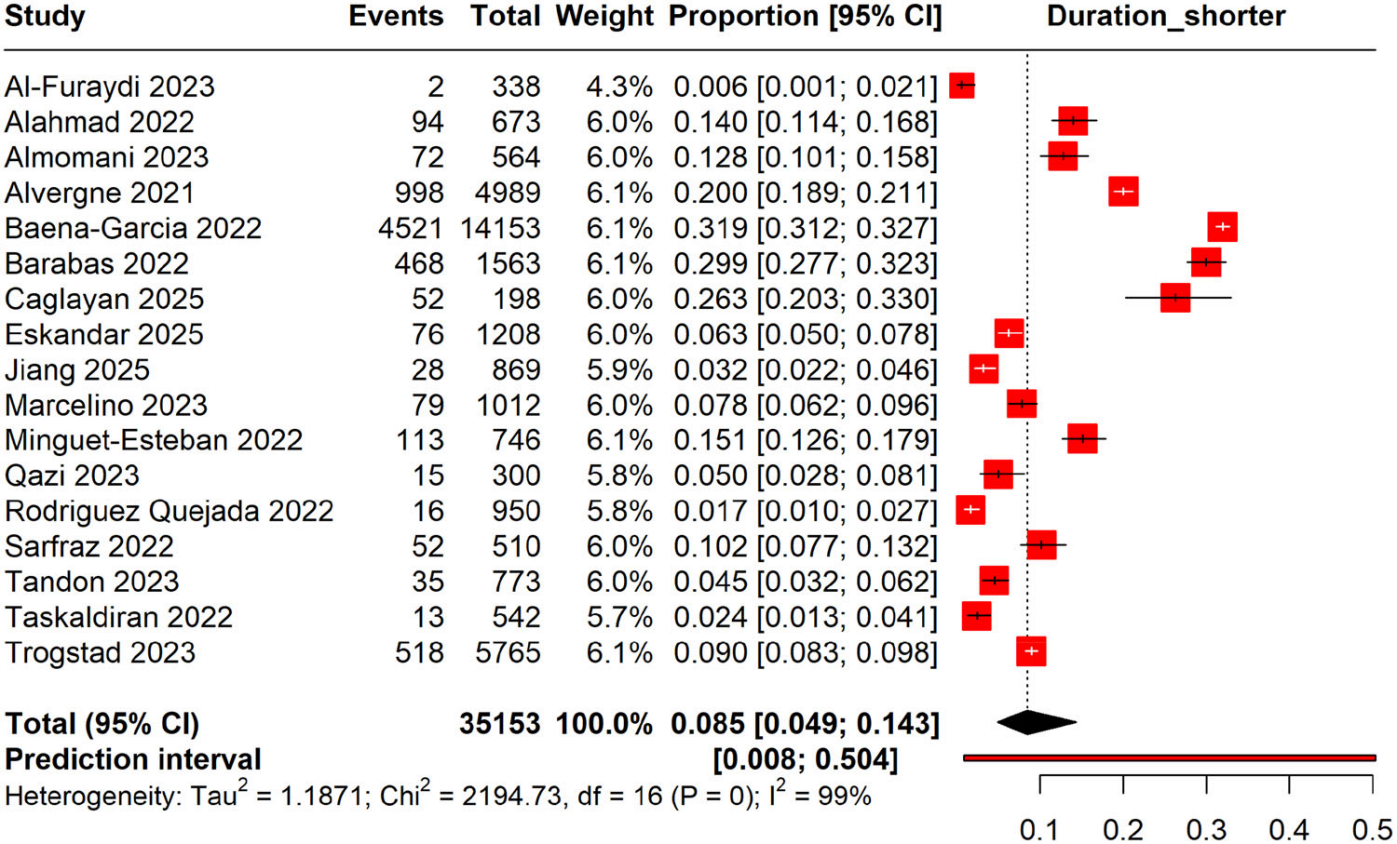
Forest plot showing the prevalence of shorter menstrual cycles following COVID‐19 vaccination.

The meta‐analysis, comprising data from 35,153 vaccinated women, estimated a pooled prevalence of shortened menstrual cycles at 8.5% (95% CI: 4.9%–14.3%) post‐COVID‐19 vaccination. The highest prevalence was reported in the study by Baena‐Garcia et al. [36] at 31.9% (95% CI: 31.2%–32.7%), while the lowest was noted in Al‐Furaydi et al. [6] at 0.6% (95% CI: 0.1%–2.1%). Heterogeneity among the included studies was high (*I*² = 99%), indicating significant variability, potentially due to differences in populations, study designs, and criteria for assessing menstrual cycle changes. The prediction interval ranged from 0.8% to 50.4%, suggesting substantial variation may be observed in future similar studies (Figure 8).

#### 3.7.2 Longer Menstruation Cycles Following COVID‐19 Vaccination

**Figure 9:**
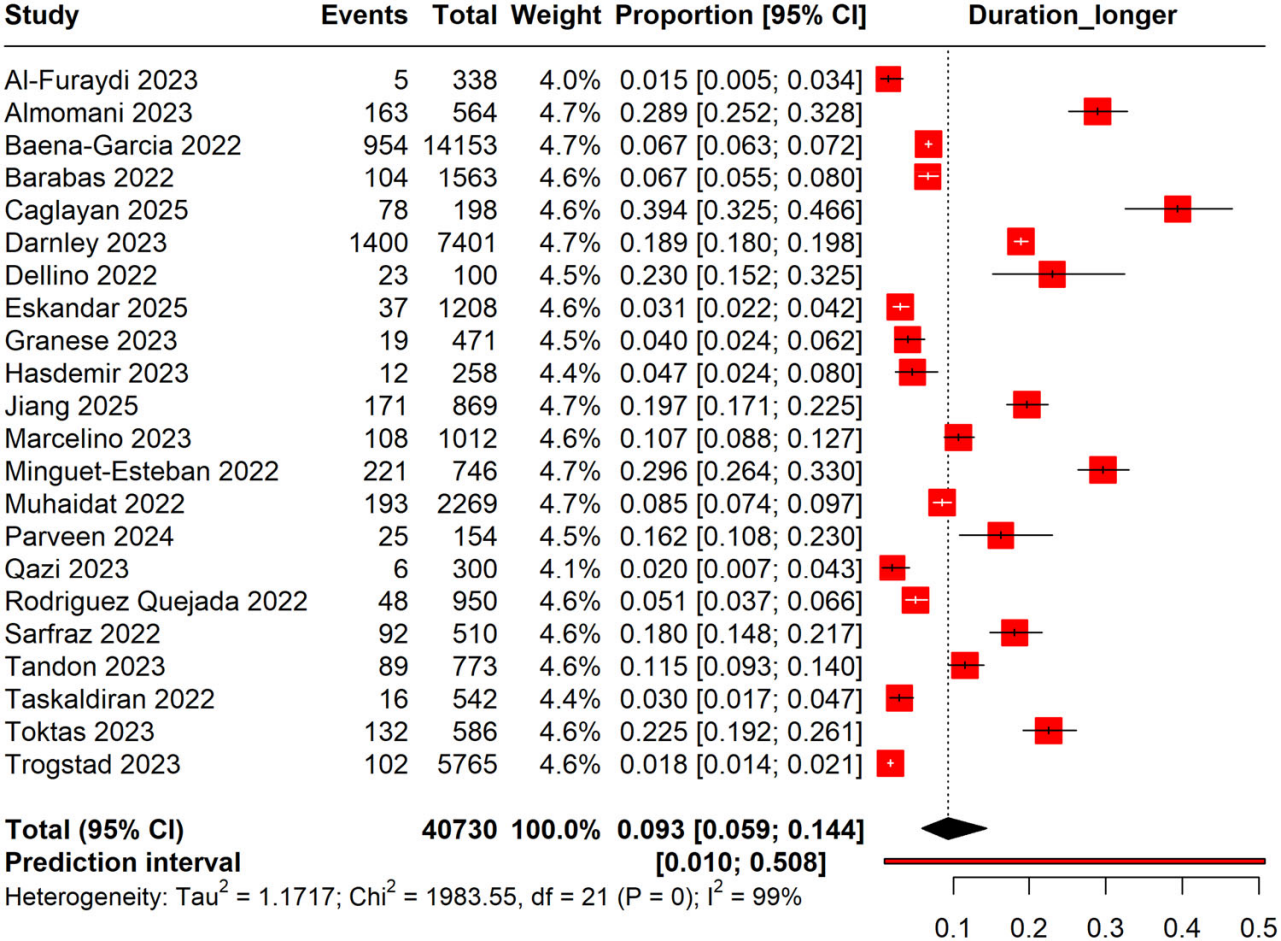
Forest plot represents the prevalence of longer menstrual cycles following COVID‐19 vaccination.

The forest plot indicated a pooled prevalence of longer menstruation cycles following COVID‐19 vaccination of 9.3% (95% CI: 5.9%–14.4%) (Figure 9). There was a significant degree of heterogeneity among the studies (*I*² = 99%), reflecting substantial variability in the reported estimates. The prediction interval ranged from 1.0% to 50.8%, suggesting that the prevalence of longer menstrual cycles may vary considerably across different populations or settings.

#### 3.7.3 Amenorrhea (< 24 Days) Following COVID‐19 Vaccination

**Figure 10:**
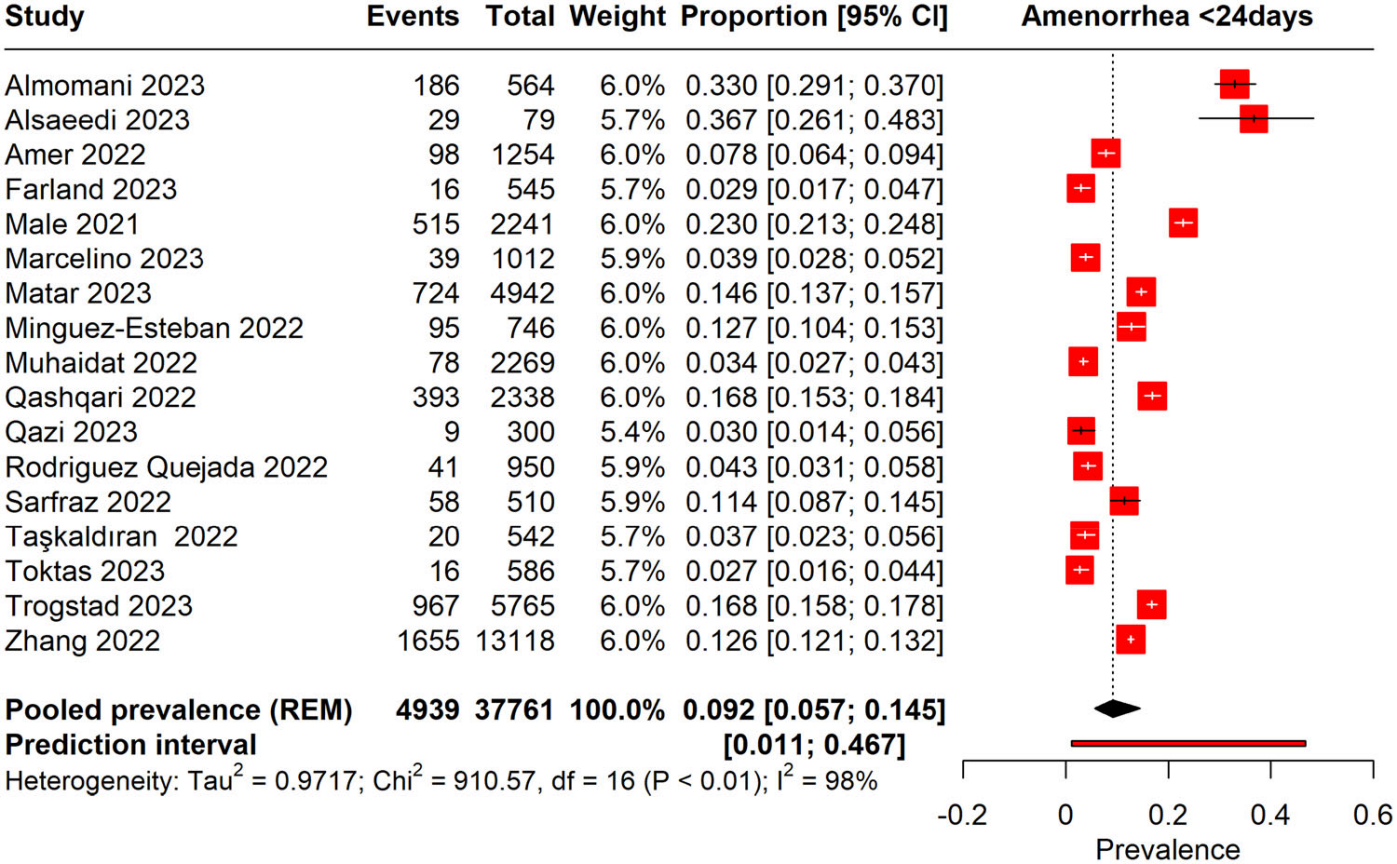
Forest plot represents the prevalence of amenorrhea of < 24 days and menstrual cycles > 38 days following COVID‐19 vaccination.

Figure 10 Forest plot assessing the prevalence of amenorrhea lasting 24 days or more following COVID‐19 vaccination. The meta‐analysis indicated a pooled prevalence of 9.2% (95% CI: 5.7%–14%). This finding suggests that a small percentage of women experienced extended amenorrhea after receiving the COVID‐19 vaccine. The high heterogeneity (*I*² = 98%) across the studies indicated significant variability in the reported rates, which could be due to differences in population demographics, vaccine types, or study methodologies. The prediction interval ranged from 0.11% to 46%, reflecting potential variance in future studies.

#### 3.7.4 Infrequent Menstrual Cycle (> 38 Days) Following COVID‐19 Vaccination

**Figure 11:**
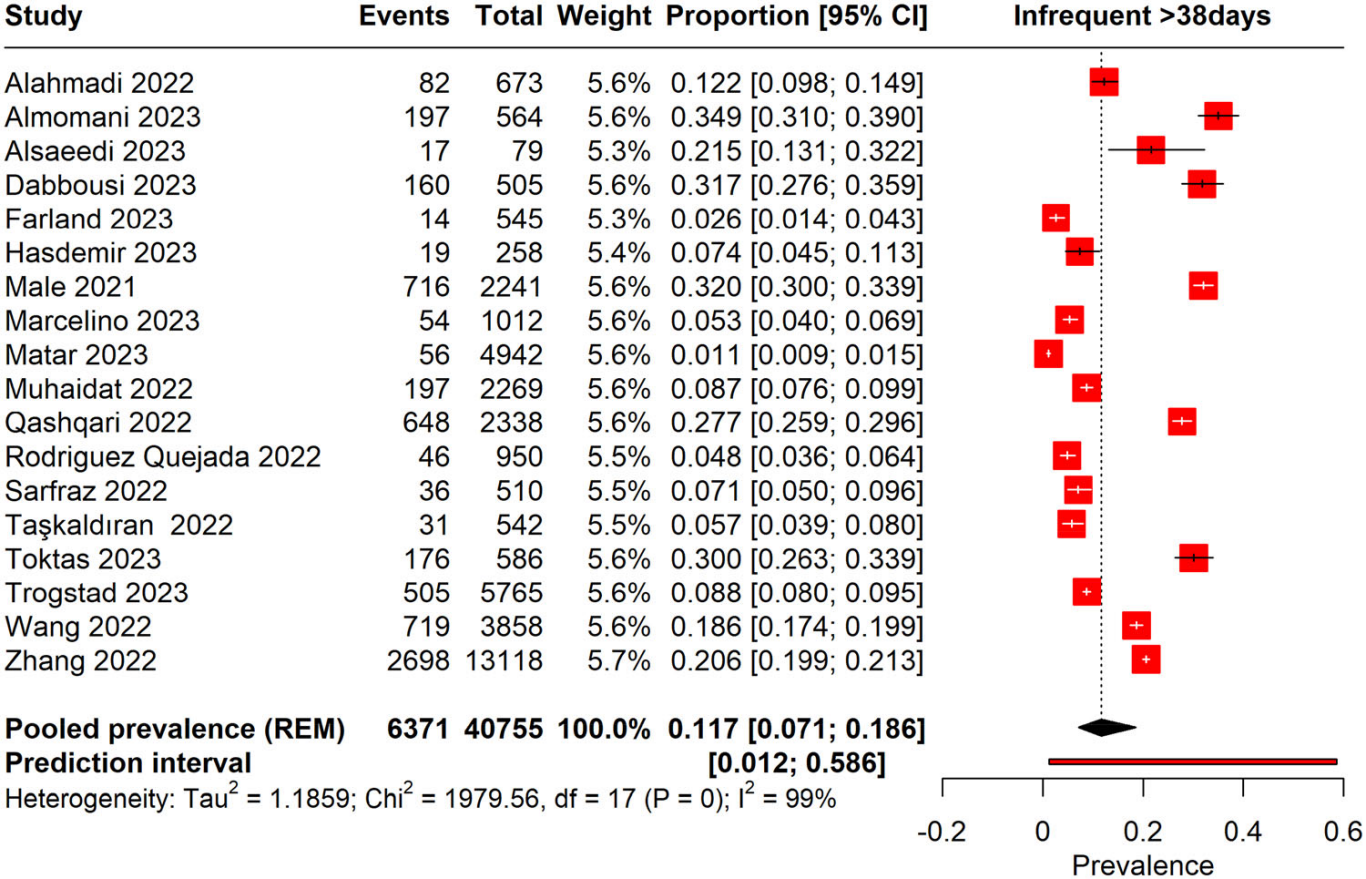
Forest plot represents the prevalence of infrequent menstrual cycles > 38 days following COVID‐19 vaccination.

The forest plot assessed the prevalence of infrequent menstruation cycles longer than 38 days after the COVID‐19 vaccination. The analysis indicated a pooled prevalence of 11% (95% CI: 7.1%–18.6%) (Figure 11). This finding suggests that a moderate percentage of women experienced less frequent menstruation after vaccination. High heterogeneity was present (*I*² = 99%), reflecting variability across different studies. The prediction interval extended from 0.12% to 58.6%, indicating that future studies could observe a wide range of prevalence rates for infrequent menstruation after vaccination.

#### 3.7.5 Sensitivity Analysis

The leave‐one‐out sensitivity analysis demonstrated consistency in the association between menstrual disturbances and COVID‐19 vaccination. The removal of individual studies did not significantly alter the overall effect size, emphasizing the robustness of the meta‐analysis findings despite high heterogeneity (*I*² = 98%) (Supporting Information S1: Figure S1). A similar analysis of the pooled prevalence revealed a stable rate of irregular menstruation after post‐COVID‐19 vaccination. Excluding individual studies led to minimal variability with slightly fluctuating prevalence rates, suggesting that no single study substantially influenced the overall prevalence rate (Supporting Information S1: Figure S2).

#### 3.7.6 Publication Bias

Egger's test indicated a *p* < 0.03, suggesting funnel plot asymmetry and potential publication bias in the studies analyzed, warranting further exploration (as depicted in Supporting Information S1: Figure S3).

## 4 Discussion

### 4.1 Main Findings

This systematic review and meta‐analysis thoroughly examined the relationship between COVID‐19 vaccination and reproductive health outcomes in women; with a particular emphasis on menstrual cycle disturbances, various menstrual changes, including irregular bleeding patterns (both lighter and heavier than usual), alterations in the regularity and duration of menstrual cycles, and instances of amenorrhea or infrequent cycles postvaccination. Despite the high heterogeneity across studies, our findings suggest nonsignificant increase in the risk of menstrual disturbances after vaccination. Importantly, the vast majority of women retained regular menstrual cycles postvaccination, indicating that COVID‐19 vaccines had a minimal impact on the overall menstrual health of the vaccinated individuals.

The pooled prevalence of menstrual disturbances was 34%, highlighting its incidence spectrum. However, when comparing the vaccinated women to the control group that did not receive the vaccine, we observed a nonsignificant increase in the risk of menstrual disturbances, with a pooled RR of 1.026. This suggests that, while vaccination may be associated with menstrual changes, the overall risk increase is minimal. Further scrutiny of the vaccinated cohort revealed a segment that experienced irregular, lighter bleeding post‐vaccination. This finding indicates the potential of the vaccine to induce minor alterations in menstrual bleeding, although such effects are neither widespread nor uniform across individuals. Concurrently, our analysis detected a prevalence of heavier menstrual bleeding among vaccinated individuals, reinforcing the notion of vaccine‐related menstrual changes. Importantly, the majority of vaccinated women reported no change in the regularity of their menstrual cycles, reinforcing the notion that COVID‐19 vaccines, for the most part, do not adversely affect menstrual cycle regularity. Notably, the prevalence of irregular menstruation after vaccination was significant, suggesting that a subset of women experienced changes in their menstrual patterns following vaccination. The findings from our analysis provide significant insights into the impact of COVID‐19 vaccination on menstrual health, a concern that has been widely discussed in both public and scientific domains. Our research, centered on the prevalence and nature of menstrual disturbances following vaccination, indicates a nuanced but generally minimal impact on menstrual health. This aligns with the primary research question aimed at understanding the extent to which COVID‐19 vaccines affect the menstrual cycle and its disturbances.

### 4.2 Strengths and Limitations

The strengths of our systematic review and meta‐analysis lie in its comprehensive search strategy and the robustness of the employed meta‐analytical methods. By including a significant number of studies, we were able to pool a diverse range of data, thus enhancing the reliability and generalizability of our findings. However, our study had some limitations. Notably, potential publication bias and heterogeneity among study designs pose challenges to the unequivocal acceptance of our findings. The variation in quality among the included studies may have influenced the overall outcomes, thereby affecting the strength of the conclusions.

Our findings suggest that, while COVID‐19 vaccination may lead to minor menstrual disturbances, these effects are generally mild and transient, offering reassurance to healthcare practitioners and their patients. Practitioners should be prepared to discuss these potential side effects and provide evidence‐based reassurance that the benefits of vaccination outweigh the risks of minor menstrual irregularities. For policymakers and public health officials, these results underscore the importance of including nuanced information on the potential side effects in vaccination guidelines and public health messaging, thereby maintaining transparency and trust in vaccination efforts.

### 4.3 Interpretation

When juxtaposed with the existing literature [32, 37, 55, 62, 64], our results echo the findings of several relevant studies that also report minor menstrual irregularities postvaccination. For instance, similar research has identified transient changes in menstrual patterns, reinforcing the notion that COVID‐19 vaccines might lead to slight alterations in menstrual health, albeit temporarily, and without significant health repercussions. The consistency between our findings and those of previous studies underscores the reliability of the emerging consensus regarding the safety profile of COVID‐19 vaccines in women's reproductive health.

However, there are discrepancies between our findings and those of previous studies that reported either no change or a higher incidence of menstrual disturbances. These variations can largely be attributed to differences in the study methodologies, population demographics, and definitions of menstrual disturbances. For example, studies utilizing self‐reported data may encounter biases or variations in participants' perceptions of menstrual changes, data collection, and interpretation [67, 68]. Additionally, the diverse geographical and demographic contexts of the studies could contribute to the observed discrepancies, as factors such as stress, lifestyle, and health status play significant roles in menstrual health and may influence how individuals respond to vaccination [51].

This review highlights the need for additional research in the area of menstrual changes post‐COVID‐19 vaccination, with a particular focus on employing standardized definitions and methodologies. Such research is crucial for updating clinical guidelines and vaccination policies using the latest and most comprehensive evidence. Given the strong safety profile of COVID‐19 vaccines, our findings reaffirm the importance of continuing vaccination efforts, which play a key role in controlling the pandemic while also monitoring for potential side effects. Improving research methodologies and standardizing outcome measures are key to a deeper understanding of how the COVID‐19 vaccination affects menstrual health. Investigating the biological mechanisms that may cause these menstrual changes is also essential, as it could shed light on the interaction between vaccines and the reproductive system. In the context of global vaccination efforts, it is paramount to address these research gaps through rigorous, high‐quality studies to ensure that vaccination policies are based on detailed and accurate data. Future research should focus on overcoming the limitations of current studies by adopting more uniform and standardized data collection methods across diverse populations and exploring the biological reasons behind the observed menstrual changes after COVID‐19 vaccination. This information will inform clinical guidelines and vaccination policies.

## 5 Conclusion

Our systematic review and meta‐analysis revealed that while COVID‐19 vaccination is associated with a notable prevalence of menstrual disturbances, there is no significant overall effect on menstrual health compared to the control group. Minor changes, such as irregular, lighter, or heavier bleeding, were observed in some vaccinated individuals; however, these effects were not consistent across the population. The overall impact of vaccination on menstrual health was minimal. This study highlights the importance of including women's reproductive health in vaccine safety assessments and emphasizes the need for continued monitoring of menstrual outcomes following vaccination.

## Author Contributions

**Ganesh Bushi:** methodology, software, and formal analysis. **Abhay M. Gaidhane:** conceptualization and validation. **Nasir Vadia:** validation, formal analysis, and project administration. **Soumya V. Menon:** methodology and conceptualization. **Kattela Chennakesavulu:** conceptualization, formal analysis, and project administration. **Rajashree Panigrahi:** software, supervision, and resources. **Muhammed Shabil:** visualization, writing – review and editing. **Diptismita Jena:** writing – review and editing, project administration. **Harish Kumar:** conceptualization, visualization, and project administration. **Anju Rani:** writing – original draft, writing – review and editing, project administration. **Sanjit Sah:** writing – review and editing, visualization, and project administration. **Shivam Rohilla:** methodology and project administration. **Mahendra Pratap Singh:** conceptualization, methodology, and formal analysis. **Khang Wen Goh:** methodology, writing – review and editing.

## Conflicts of Interest

The authors declare no conflicts of interest.

## Transparency Statement

The lead authors, Ganesh Bushi and Abhay M. Gaidhane, affirm that this manuscript is an honest, accurate, and transparent account of the study being reported; that no important aspects of the study have been omitted; and that any discrepancies from the study as planned (and, if relevant, registered) have been explained.

## Supporting information

Supplementary R1.

## Data Availability

All data generated or analyzed during this study are included in this article (and Supporting Information S1).

## References

[1] K. Bok , S. Sitar , B. S. Graham , and J. R. Mascola , “Accelerated COVID‐19 Vaccine Development: Milestones, Lessons, and Prospects,” Immunity 54, no. 8 (2021): 1636–1651. 34348117 10.1016/j.immuni.2021.07.017 PMC8328682

[2] H. Yarlagadda , M. A. Patel , V. Gupta , et al., “COVID‐19 Vaccine Challenges in Developing and Developed Countries,” Cureus 14, no. 4 (2022): e23951. 35547442 10.7759/cureus.23951 PMC9085716

[3] E. Mathieu , H. Ritchie , L. Rodés‐Guirao , et al., “Coronavirus Pandemic (COVID‐19),” Our World in Data (2020).

[4] M. Shabil , S. Gaidhane , S. Ballal , et al., “Maternal COVID‐19 Infection and Risk of Respiratory Distress Syndrome Among Newborns: A Systematic Review and Meta‐Analysis,” BMC Infectious Diseases 24, no. 1 (2024): 1318. 39563236 10.1186/s12879-024-10161-1 PMC11577808

[5] F. Chirico , J. A. Teixeira da Silva , P. Tsigaris , and K. Sharun , “Safety & Effectiveness of COVID‐19 Vaccines: A Narrative Review,” Indian Journal of Medical Research 155, no. 1 (2022): 91–104. 35859436 10.4103/ijmr.IJMR_474_21 PMC9552389

[6] A. Al‐Furaydi , S. A. Alrobaish , and N. Al‐Sowayan , “The COVID‐19 Vaccines and Menstrual Disorders,” European Review for Medical and Pharmacological Sciences 27, no. 3 (2023): 1185–1191. 36808367 10.26355/eurrev_202302_31225

[7] E. Y. Almomani , R. Hajjo , A. Qablan , D. A. Sabbah , and A. Al‐Momany , “A Cross‐Sectional Study Confirms Temporary Post‐COVID‐19 Vaccine Menstrual Irregularity and the Associated Physiological Changes Among Vaccinated Women in Jordan,” Frontiers in Medicine 10 (2023): 1211283. 37869161 10.3389/fmed.2023.1211283 PMC10587412

[8] M. Jaffry , F. Mostafa , K. Mandava , et al., “No Significant Increase in Guillain‐Barré Syndrome After COVID‐19 Vaccination in Adults: A Vaccine Adverse Event Reporting System Study,” Vaccine 40, no. 40 (2022): 5791–5797. 36055875 10.1016/j.vaccine.2022.08.038 PMC9393181

[9] I. Caplanusi , A. Szmigiel , M. van der Elst , et al., “The Role of the European Medicines Agency in the Safety Monitoring of COVID‐19 Vaccines and Future Directions in Enhancing Vaccine Safety Globally,” Drug Safety 47, no. 5 (2024): 405–418. 38396269 10.1007/s40264-024-01405-9 PMC11018685

[10] M. Gordillo‐Marañón , A. Szmigiel , V. Yalmanová , et al., “COVID‐19 Vaccines and Heavy Menstrual Bleeding: The Impact of Media Attention on Reporting to Eudravigilance,” Drug Safety 47, no. 8 (2024): 783–798. 38607521 10.1007/s40264-024-01426-4 PMC11286647

[11] A. Arshad , M. Usman , H. Majeed Khan , Z. Rahman , M. Manzoor , and B. Gul , “Effect of COVID‐19 Vaccination on Menstrual Cycle Irregularities Among Females: A Cross‐Sectional Study in Pakistan,” Pakistan Journal of Pharmaceutical Sciences 37 (2024): 215–222. 38747272

[12] S. G. Matar , A. Z. Nourelden , A. Assar , et al., “Effect of COVID‐19 Vaccine on Menstrual Experience Among Females in Six Arab Countries: A Cross Sectional Study,” Influenza and Other Respiratory Viruses 17, no. 1 (2023): e13088. 36578138 10.1111/irv.13088 PMC9835440

[13] F. Peinemann , D. Oberle , U. Drechsel‐Bäuerle , and B. Keller‐Stanislawski , “Adverse Menstrual Events Reported After and Before (or Without) COVID‐19 Vaccination: A Systematic Review and Meta‐Analysis of Comparative Observational Studies,” Pharmacoepidemiology and Drug Safety 33, no. 8 (2024): e5877. 39090813 10.1002/pds.5877

[14] K. K. Wong , C. M. Heilig , A. Hause , et al., “Menstrual Irregularities and Vaginal Bleeding After COVID‐19 Vaccination Reported to V‐Safe Active Surveillance, USA in December, 2020–January, 2022: An Observational Cohort Study,” Lancet Digital Health 4, no. 9 (2022): e667–e675. 35961858 10.1016/S2589-7500(22)00125-X PMC9363036

[15] X. Zheng , Y. Fang , Y. Song , et al., “Is There a Causal Nexus Between COVID‐19 Infection, COVID‐19 Vaccination, and Guillain‐Barré Syndrome?,” European Journal of Medical Research 28, no. 1 (2023): 98. 36841799 10.1186/s40001-023-01055-0 PMC9958317

[16] R. Wali , H. Alhindi , A. Saber , K. Algethami , and R. Alhumaidah , “The Effect of COVID‐19 Vaccine on Women's Reproductive Health: A Cross‐Sectional Study,” Cureus 15, no. 6 (2023): e40076. 37425538 10.7759/cureus.40076 PMC10326796

[17] G. Bushi , S. Gaidhane , S. Ballal , et al., “Postural Orthostatic Tachycardia Syndrome After COVID‐19 Vaccination: A Systematic Review,” BMC Cardiovascular Disorders 24, no. 1 (2024): 643. 39538129 10.1186/s12872-024-04315-x PMC11562304

[18] C. Wang , M. Wang , G. Li , B. Song , Q. Xing , and Y. Cao , “Effects of COVID‐19 Vaccination on Human Fertility: A Post‐Pandemic Literature Review,” Annals of Medicine 55, no. 2 (2023): 2261964. 37756386 10.1080/07853890.2023.2261964 PMC10538453

[19] V. R. Smaardijk , R. Jajou , A. Kant , and F. P. A. M. van Hunsel , “Menstrual Disorders Following COVID‐19 Vaccination: A Review Using a Systematic Search,” Frontiers in Drug Safety and Regulation 4 (2024): 1338466. 10.3389/fdsfr.2024.1338466 PMC12443100 40979395

[20] L. A. Payne , L. A. Wise , A. K. Wesselink , S. Wang , S. A. Missmer , and A. Edelman , “Association Between COVID‐19 Vaccination and Menstruation: A State of the Science Review,” BMJ Sexual & Reproductive Health 50, no. 3 (2024): 212–225. 38857991 10.1136/bmjsrh-2024-202274 PMC11246222

[21] C. K.‐L. Lo , D. Mertz , and M. Loeb , “Newcastle‐Ottawa Scale: Comparing Reviewers' to Authors' Assessments,” BMC Medical Research Methodology 14 (2014): 1–5. 24690082 10.1186/1471-2288-14-45 PMC4021422

[22] M. A. Shamim , A. P. Gandhi , P. Dwivedi , and B. K. Padhi , “How to Perform Meta‐Analysis in R: A Simple yet Comprehensive Guide,” Evidence 1, no. 1 (2023): 60–80.

[23] D. Langan , J. P. T. Higgins , D. Jackson , et al., “A Comparison of Heterogeneity Variance Estimators in Simulated Random‐Effects Meta‐Analyses,” Research Synthesis Methods 10, no. 1 (2019): 83–98. 30067315 10.1002/jrsm.1316

[24] A. P. Gandhi , M. A. Shamim , and B. K. Padhi , “Steps in Undertaking Meta‐Analysis and Addressing Heterogeneity in Meta‐Analysis,” Evidence 1, no. 1 (2023): 44–59.

[25] M. Shabil , G. Bushi , and M. N. Khatib , “A Commentary on ‘Psychological Health Among Healthcare Professionals During COVID‐19 Pandemic: An Updated Meta‐Analysis’,” Indian Journal of Psychiatry 66, no. 8 (2024): 763–764. 39398520 10.4103/indianjpsychiatry.indianjpsychiatry_496_24 PMC11469564

[26] K. Barabas , B. Makkai , N. Farkas , et al., “Influence of COVID‐19 Pandemic and Vaccination on the Menstrual Cycle: A Retrospective Study in Hungary,” Frontiers in Endocrinology 13 (2022): 974788. 36387878 10.3389/fendo.2022.974788 PMC9646704

[27] M. Dellino , B. Lamanna , M. Vinciguerra , et al., “SARS‐CoV‐2 Vaccines and Adverse Effects in Gynecology and Obstetrics: The First Italian Retrospective Study,” International Journal of Environmental Research and Public Health 19, no. 20 (2022): 13167. 36293746 10.3390/ijerph192013167 PMC9603573

[28] G. A. Khan , A. Althubaiti , A. Alshrif , Z. Alsayed , and H. Jifree , “Dysmenorrhea, Intermenstrual Bleeding, Menstrual Flow Volume Changes, and Irregularities Following COVID‐19 Vaccination and the Association With Vaccine Skepticism: A Retrospective Observational Study,” Women's Health 19 (2023): 17455057231210094. 10.1177/17455057231210094 PMC10652665 37966030

[29] M. C. Magnus , I. H. Caspersen , K.‐A. Wensaas , et al., “Covid‐19 Vaccination and Menstrual Bleeding Disturbances Among Women of Fertile Age: A Norwegian Registry Study,” European Journal of Epidemiology 39 (2024): 1127–1138. 39503924 10.1007/s10654-024-01170-0 PMC11599392

[30] V. Male , “Effect of COVID‐19 Vaccination on Menstrual Periods in a Retrospectively Recruited Cohort,” MedRXiv (2021): 2021.11.15.21266317 In press.

[31] L. Rodriguez Quejada , M. F. Toro Wills , M. C. Martinez‐Avila , and A. F. Patino‐Aldana , “Menstrual Cycle Disturbances After COVID‐19 Vaccination,” Womens Health (London, England) 18 (2022): 17455057221109375. 10.1177/17455057221109375 PMC9295013 35796571

[32] B. Zhang , X. Yu , J. Liu , J. Liu , and P. Liu , “COVID‐19 Vaccine and Menstrual Conditions in Female: Data Analysis of the Vaccine Adverse Event Reporting System (VAERS),” BMC Women's Health 22, no. 1 (2022): 403. 36195902 10.1186/s12905-022-01934-4 PMC9532224

[33] A. M. Aljehani , S. A. Banjar , H. S. Alawam , et al., “The Relationship Between Menstrual Cycle Irregularities and COVID‐19 Vaccination,” Cureus 15, no. 12 (2023): e49841. 38164312 10.7759/cureus.49841 PMC10758269

[34] E. Y. Almomani , R. Hajjo , A. Qablan , D. A. Sabbah , and A. Al‐Momany , “A Cross‐Sectional Study Confirms Temporary post‐COVID‐19 Vaccine Menstrual Irregularity and the Associated Physiological Changes Among Vaccinated Women in Jordan,” Frontiers in Medicine 10 (2023): 1211283. 37869161 10.3389/fmed.2023.1211283 PMC10587412

[35] A. A. Amer , S. A. Amer , K. M. Alrufaidi , et al., “Menstrual Changes After COVID‐19 Vaccination and/or SARS‐CoV‐2 Infection and Their Demographic, Mood, and Lifestyle Determinants in Arab Women of Childbearing Age, 2021,” Frontiers in Reproductive Health 4 (2022): 927211. 36303671 10.3389/frph.2022.927211 PMC9580647

[36] L. Baena‐García , V. A. Aparicio , A. Molina‐López , P. Aranda , L. Cámara‐Roca , and O. Ocón‐Hernández , “Premenstrual and Menstrual Changes Reported After COVID‐19 Vaccination: The EVA Project,” Women's Health 18 (2022): 17455057221112237. 10.1177/17455057221112237 PMC9289916 35833668

[37] A. A. Dabbousi , J. El Masri , L. M. El Ayoubi , O. Ismail , B. Zreika , and P. Salameh , “Menstrual Abnormalities Post‐Covid Vaccination: A Cross‐Sectional Study on Adult Lebanese Women,” Irish Journal of Medical Science 192, no. 3 (2023): 1163–1170. 35881229 10.1007/s11845-022-03089-5 PMC9315076

[38] M. Eskandar , A. Alassim , F. Riaz , et al., “Coronavirus Disease 2019 Vaccination and Menstrual Cycle Changes: A Cross‐Sectional Study on Females of Reproductive Age in Saudi Arabia,” Medicine 104, no. 8 (2025): e41656. 39993068 10.1097/MD.0000000000041656 PMC11856927

[39] R. Granese , G. G. Incognito , F. A. Gulino , et al., “Effects of SARS‐CoV‐2 Vaccination on Menstrual Cycle: An Italian Survey‐Based Study,” Journal of Clinical Medicine 12, no. 24 (2023): 7699. 38137768 10.3390/jcm12247699 PMC10744112

[40] G. Issakov , Y. Tzur , T. Friedman , and T. Tzur , “Abnormal Uterine Bleeding Among COVID‐19 Vaccinated and Recovered Women: A National Survey,” Reproductive Sciences 30, no. 2 (2023): 713–721. 35986194 10.1007/s43032-022-01062-2 PMC9390105

[41] Y. Jiang , Y. Li , and Y. Huang , “Alterations in Menstrual Characteristics and Associated Factors in Chinese Women Post SARS‐CoV‐2 Infection: A Cross‐Sectional Study,” BMC Women's Health 25, no. 1 (2025): 69. 39966921 10.1186/s12905-025-03592-8 PMC11837296

[42] R. Kareem , M. R. Sethi , S. Inayat , and M. Irfan , “The Effect of COVID‐19 Vaccination on the Menstrual Pattern and Mental Health of the Medical Students: A Mixed‐Methods Study From a Low and Middle‐Income Country,” PLoS One 17, no. 11 (2022): e0277288. 36355919 10.1371/journal.pone.0277288 PMC9648815

[43] M. S. Mahfouz , M. M. Abdelmageed , A. Y. Alqassim , et al., “Menstrual Irregularities Associated With COVID‐19 Vaccines Among Women in Saudi Arabia: A Survey During 2022,” Open Medicine 18, no. 1 (2023): 20230804. 37829840 10.1515/med-2023-0804 PMC10566562

[44] A. C. Marcelino , A. B. Fim , P. da Cunha Pereira , I. Monteiro , B. G. Darney , and L. Bahamondes , “Association Between COVID‐19 and Vaccination on Menstrual Cycle,” International Journal of Gynaecology and Obstetrics 164, no. 2 (2024): 571–577. 37855055 10.1002/ijgo.15200

[45] S. G. Matar , A. Z. Nourelden , A. Assar , et al., “Effect of COVID‐19 Vaccine on Menstrual Experience Among Females in Six Arab Countries: A Cross Sectional Study. Influenza and Other Respiratory,” Viruses 17, no. 1 (2023): e13088. 10.1111/irv.13088 PMC9835440 36578138

[46] I. Mínguez‐Esteban , P. García‐Ginés , C. Romero‐Morales , et al., “Association Between RNAm‐Based COVID‐19 Vaccines and Permanency of Menstrual Cycle Alterations in Spanish Women: A Cross‐Sectional Study,” Biology 11, no. 11 (2022): 1579. 36358280 10.3390/biology11111579 PMC9687584

[47] N. Muhaidat , M. A. Alshrouf , M. I. Azzam , A. M. Karam , M. Al‐Nazer , and A. Al‐Ani , “Menstrual Symptoms After COVID‐19 Vaccine: A Cross‐Sectional Investigation in the MENA Region,” International Journal of Women's Health 14 (2022): 395–404. 10.2147/IJWH.S352167 PMC8976114 35378876

[48] F. Qashqari , M. Dahlawi , H. M. Assaggaf , et al., “Effect of the COVID‐19 Vaccine on the Menstrual Cycle Among Females in Saudi Arabia,” Ethiopian Journal of Health Sciences 32, no. 6 (2022): 1083–1092. 36475264 10.4314/ejhs.v32i6.4 PMC9692149

[49] T. B. Qazi , S. A. Dkhar , R. Quansar , and S. M. S. Khan , “Impact of COVID‐19 Vaccination on Menstrual Cycle in Women of Reproductive Age,” International Journal of Gynecology & Obstetrics 162, no. 3 (2023): 1086–1090. 37132582 10.1002/ijgo.14822

[50] M. H. Safiah , K. K. Al Ashabi , N. Khalayli , Y. Hodaifa , and M. Kudsi , “The Prevalence of Menstrual Changes in COVID‐19 Vaccinated Women: A Cross‐Sectional Study,” Preventive Medicine Reports 44 (2024): 102804. 39040951 10.1016/j.pmedr.2024.102804 PMC11261097

[51] H. Saleh Alzahrani , S. Ali Algashami , A. Abdulaziz Alharkan , N. Sultan Alotaibi , and N. Waseem Algahs , “The Effect of COVID‐19 Vaccination on the Menstrual Cycle in Female in Riyadh, Saudi Arabia,” Saudi Pharmaceutical Journal 31, no. 5 (2023): 746–751. 37128295 10.1016/j.jsps.2023.03.015 PMC10063452

[52] A. Sarfraz , Z. Sarfraz , M. Sarfraz , Z. Nadeem , M. Felix , and I. Cherrez‐Ojeda , “Menstrual Irregularities Following COVID‐19 Vaccination: A Global Cross‐Sectional Survey,” Annals of Medicine & Surgery 81 (2022): 104220. 35957648 10.1016/j.amsu.2022.104220 PMC9356761

[53] M. Sualeh , M. R. Uddin , N. Junaid , M. Khan , A. Pario , and Q. Ain , “Impact of COVID‐19 Vaccination on Menstrual Cycle: A Cross‐Sectional Study From Karachi, Pakistan,” Cureus 14, no. 8 (2022): e28630. 36196308 10.7759/cureus.28630 PMC9524410

[54] A. Tandon , N. Kumar , S. Aggarwal , et al., “Assessing Menstrual Changes Among Young Indian Females Post‐SARS‐CoV‐2 Vaccination,” Cureus 15, no. 12 (2023): e50025. 38186546 10.7759/cureus.50025 PMC10767692

[55] I. Taskaldiran , E. Vuraloglu , Y. Bozkus , O. Turhan Iyidir , A. Nar , and N. Bascil Tutuncu , “Menstrual Changes After COVID‐19 Infection and COVID‐19 Vaccination,” International Journal of Clinical Practice 2022 (2022): 3199758. 36349056 10.1155/2022/3199758 PMC9633189

[56] I. Toktaş , H. Akelma , and E. Araç , “Examining the Effect of COVID‐19 Vaccines on the Menstrual Cycle: A Study From Turkey,” Medicine 102, no. 50 (2023): e36638. 38115291 10.1097/MD.0000000000036638 PMC10727619

[57] F. A. Alsaeedi , A. F. Gharib , A. F. Hassan , et al., “Influence of COVID‐19 Infection/Vaccination on Menstrual Regularity and Hormonal Function in Saudi Females of Reproductive Age,” Heliyon 9, no. 11 (2023): e22291. 38058640 10.1016/j.heliyon.2023.e22291 PMC10695999

[58] A. Alvergne , G. Kountourides , M. A. Argentieri , et al., “A Retrospective Case‐Control Study on Menstrual Cycle Changes Following COVID‐19 Vaccination and Disease,” iScience 26, no. 4 (2023): 106401. 36987520 10.1016/j.isci.2023.106401 PMC10015085

[59] I. S. C. Caglayan , G. Demirel , and C. E. Can , “Associations Between COVID‐19 Vaccination Status and Persistent Symptoms: A Prospective Study of Reproductive‐Age Women,” Journal of Evaluation in Clinical Practice 31, no. 1 (2025): e70005. 39918012 10.1111/jep.70005

[60] B. G. Darney , E. R. Boniface , A. Van Lamsweerde , et al., “Impact of Coronavirus Disease 2019 (COVID‐19) Vaccination on Menstrual Bleeding Quantity: An Observational Cohort Study,” BJOG: An International Journal of Obstetrics & Gynaecology 130, no. 7 (2023): 803–812. 37035899 10.1111/1471-0528.17471

[61] L. V. Farland , S. M. Khan , A. Shilen , et al., “COVID‐19 Vaccination and Changes in the Menstrual Cycle Among Vaccinated Persons,” Fertility and Sterility 119, no. 3 (2023): 392–400. 36539055 10.1016/j.fertnstert.2022.12.023 PMC9758067

[62] P. S. Hasdemir , S. Senol Akar , A. Goker , et al., “The Effect of COVID‐19 Vaccinations on Menstrual Cycle and Serum Anti‐Mullerian Hormone Levels in Reproductive Age Women,” Human Fertility 26, no. 1 (2023): 153–161. 36919413 10.1080/14647273.2023.2181710

[63] N. Parveen , “COVID‐19 Vaccination and Menstrual Disturbances: A Prospective Study From Pakistan,” Pakistan Journal of Medical Sciences 40, no. 7 (2024): 1345. 39092064 10.12669/pjms.40.7.8709 PMC11255808

[64] L. Trogstad , I. Laake , A. H. Robertson , et al., “Heavy Bleeding and Other Menstrual Disturbances in Young Women After COVID‐19 Vaccination,” Vaccine 41, no. 36 (2023): 5271–5282. 37451876 10.1016/j.vaccine.2023.06.088

[65] S. Wang , J. Mortazavi , J. E. Hart , et al., “A Prospective Study of the Association Between SARS‐CoV‐2 Infection and COVID‐19 Vaccination With Changes in Usual Menstrual Cycle Characteristics,” American Journal of Obstetrics and Gynecology 227, no. 5 (2022): 739.e1–739.e11. 10.1016/j.ajog.2022.07.003 PMC9277995 35841938

[66] A. M. Alahmadi , A. H. Aljohani , R. A. Fadhloun , A. S. Almohammadi , D. F. Alharbi , and L. S. Alrefai , “The Effect of the COVID‐19 Vaccine on the Menstrual Cycle Among Reproductive‐Aged Females in Saudi Arabia,” Cureus 14, no. 12 (2022): e32473. 36523858 10.7759/cureus.32473 PMC9745981

[67] L. Trogstad , L. Juvet , B. Feiring , and K. Blix , “Covid‐19 Vaccines and Menstrual Changes,” BMJ Medicine 1, no. 1 (2022): e000357. 36936587 10.1136/bmjmed-2022-000357 PMC9951358

[68] P. Satapathy , A. M. Gaidhane , N. Vadia , et al., “Impact of SGLT‐2i on COPD Exacerbations in Patients With Type 2 Diabetes Mellitus: A Systematic Review and Meta‐Analysis,” Diabetes & Metabolism 51 (2025): 101646. 40220861 10.1016/j.diabet.2025.101646

